# Hepatic Arterial Infusion Chemotherapy of Oxaliplatin, Fluorouracil, and Leucovorin With or Without Sorafenib as Initial Treatment for Advanced Hepatocellular Carcinoma

**DOI:** 10.3389/fonc.2021.619461

**Published:** 2021-05-12

**Authors:** Run-Bin Liang, Yang Zhao, Min-Ke He, Dong-Sheng Wen, Xiao-Yun Bu, Ye-Xing Huang, Zhi-Cheng Lai, Yu-Jie Xu, Anna Kan, Wei Wei, Yao-Jun Zhang, Min-Shan Chen, Rong-Ping Guo, Qi-Jiong Li, Ming Shi

**Affiliations:** Department of Hepatobiliary Oncology, Sun Yat-sen University Cancer Center, State Key Laboratory of Oncology in South China, Collaborative Innovation Center for Cancer Medicine, Guangzhou, China

**Keywords:** hepatocellular carcinoma, hepatic arterial infusion chemotherapy, FOLFOX, sorafenib, treatment

## Abstract

**Purpose:**

Our previous study showed that hepatic arterial infusion chemotherapy (HAIC) using oxaliplatin, fluorouracil, and leucovorin (FOLFOX) plus sorafenib provided a significant survival benefit over sorafenib for advanced hepatocellular carcinoma. However, it is unclear whether the survival benefit should be attributed to the synergism between HAIC and sorafenib or just HAIC alone. We aim to compare HAIC using FOLFOX plus sorafenib with HAIC alone in patients with advanced hepatocellular carcinoma.

**Materials and Methods:**

This was a retrospective study including 225 eligible patients treated with HAIC using FOLFOX (HAIC alone group, n=126, oxaliplatin 85 mg/m², leucovorin 400 mg/m², fluorouracil bolus 400 mg/m² and 2400 mg/m² for 46 hours, every 3 weeks) alone or HAIC plus sorafenib (soraHAIC group, n=99, sorafenib 400 mg twice daily). Survival curves were calculated by the Kaplan-Meier method, and propensity-score matching was used to reduce bias.

**Results:**

The soraHAIC group showed a longer overall survival (12.9 [95% CI, 10.4-15.4] vs. 10.5 [95% CI, 9.5-11.5] months, HR=0.71 [95% CI, 0.53-0.96]; *P*=0.025), a better progression free survival (7.0 [95% CI, 5.3-8.8] vs. 5.3 [95% CI, 3.5-7.1] months, HR=0.76 [95% CI, 0.58-0.99]; *P*=0.046), and a higher disease control rate (RECIST 1.1: 74.8% vs. 61.1%, *P*=0.030) than the HAIC alone group. In multivariate analysis, soraHAIC was an independent favor factor for survival. In terms of the grade 3/4 adverse event, hand–foot skin reaction was more frequent in the soraHAIC group than the HAIC alone group. In the propensity-score matched cohorts (93 pairs), the overall survival, the progression free survival and disease control rates in the soraHAIC group were also better than those in the HAIC group (*P*<0.05).

**Conclusion:**

HAIC plus sorafenib may improve overall survival and progression free survival compared with HAIC alone as initial treatment for advanced hepatocellular carcinoma.

## Introduction

Hepatocellular carcinoma (HCC) is one of the most common human malignancies in the world, ranking as the second leading cause of cancer-related death (1). Approximately 50% of patients are diagnosed with macroscopic vascular invasion or distant metastasis (advanced stage), and curative treatments, such as surgical resection, ablation and liver transplantation, are not applicable (2, 3). The traditional standard treatment for these patients is sorafenib, which has suffered from high-level heterogeneity of individual response (4) and median overall survival time (OS) of only 6.5-10.7 months (5, 6).

As an alternative therapy to sorafenib, hepatic arterial infusion chemotherapy (HAIC) is recommended for advanced HCC in Japan (7), with high response rates, favorable long-term outcomes, and acceptable toxicities (8, 9). In 2018, a large sample retrospective study showed that hepatic arterial infusion of oxaliplatin, fluorouracil, and leucovorin (FOLFOX) monotherapy can significantly improve survival compared with sorafenib for advanced HCC (median OS 14.5 vs. 7.0 months; *P*<0.001) (10). Recently, our previous randomized phase 3 trial have also demonstrated that hepatic arterial infusion of FOLFOX plus sorafenib provided marked survival benefits over sorafenib for advanced HCC (median OS 13.37 vs. 7.13 months; *P*<0.001) (11).

However, previous studies have failed to show whether the prolonged survival benefit should be attributed to the synergism between HAIC with FOLFOX and sorafenib or just HAIC alone (10, 11). HAIC with cisplatin plus sorafenib in phase II trials has shown favorable OS and a manageable safety profile (12), though in phase III trials did not (13). Some previous studies also suggested that sorafenib might increase the platinum sensitivity thus exert a synergistic anticancer effect (14–16), while others suggested that sorafenib might reduce cellular uptake of platinum compounds and cytotoxicity thus exert an antagonistic effect (17, 18). Therefore, research to date has not yet determined whether sorafenib plus HAIC is superior to HAIC alone.

To answer this question, we conducted a retrospective study to compare the efficacy and safety of HAIC using FOLFOX plus sorafenib to HAIC alone in patients with advanced HCC. We hope that our data will make some contribution to fill the gap in the literature.

## Materials and Methods

This was a retrospective study performed in China. From March 1, 2016 to July 22, 2018, 225 consecutive HCC patients with HAIC alone or HAIC plus sorafenib as initial treatment were included in this study. The study was approved by the Institutional Review Board of Sun Yat-sen University Cancer Center (no. 5010-2018-06-01) and was conducted in accordance with the Declaration of Helsinki.

### Patients Selection

The inclusion criteria were as follows: HCC patients in stage C according to the Barcelona Clinic Liver Cancer staging system (19); Child-Pugh class A; an Eastern Cooperative Oncology Group performance status (ECOG PS) of 0-2; no previous treatment for HCC; at least one measurable lesion according to the Response Evaluation Criteria in Solid Tumors (RECIST) version 1.1 (20); at least a cycle of HAIC; and adequate organ function (leukocyte count ≥3.0×10^9^/L, absolute neutrophils ≥1.5×10^9^/L, platelet cell count ≥75×10^9^/L, albumin ≥30 g/L, total bilirubin ≤30 μmol/L, and transaminase ≤ 5 times the upper limit of the normal range).

The exclusion criteria consisted of the following: hepatic decompensation according to the European Association for the Study of the Liver guidelines (21); combination with other treatments, including transarterial chemoembolization (TACE), lenvatinib, immune checkpoint inhibitors, radiotherapy, and systemic chemotherapy; human immunodeficiency virus infection; pregnancy or breastfeeding; a second malignancy; loss to follow-up; and lack of imaging prior to the initiation of the treatments.

**Figure 1:**
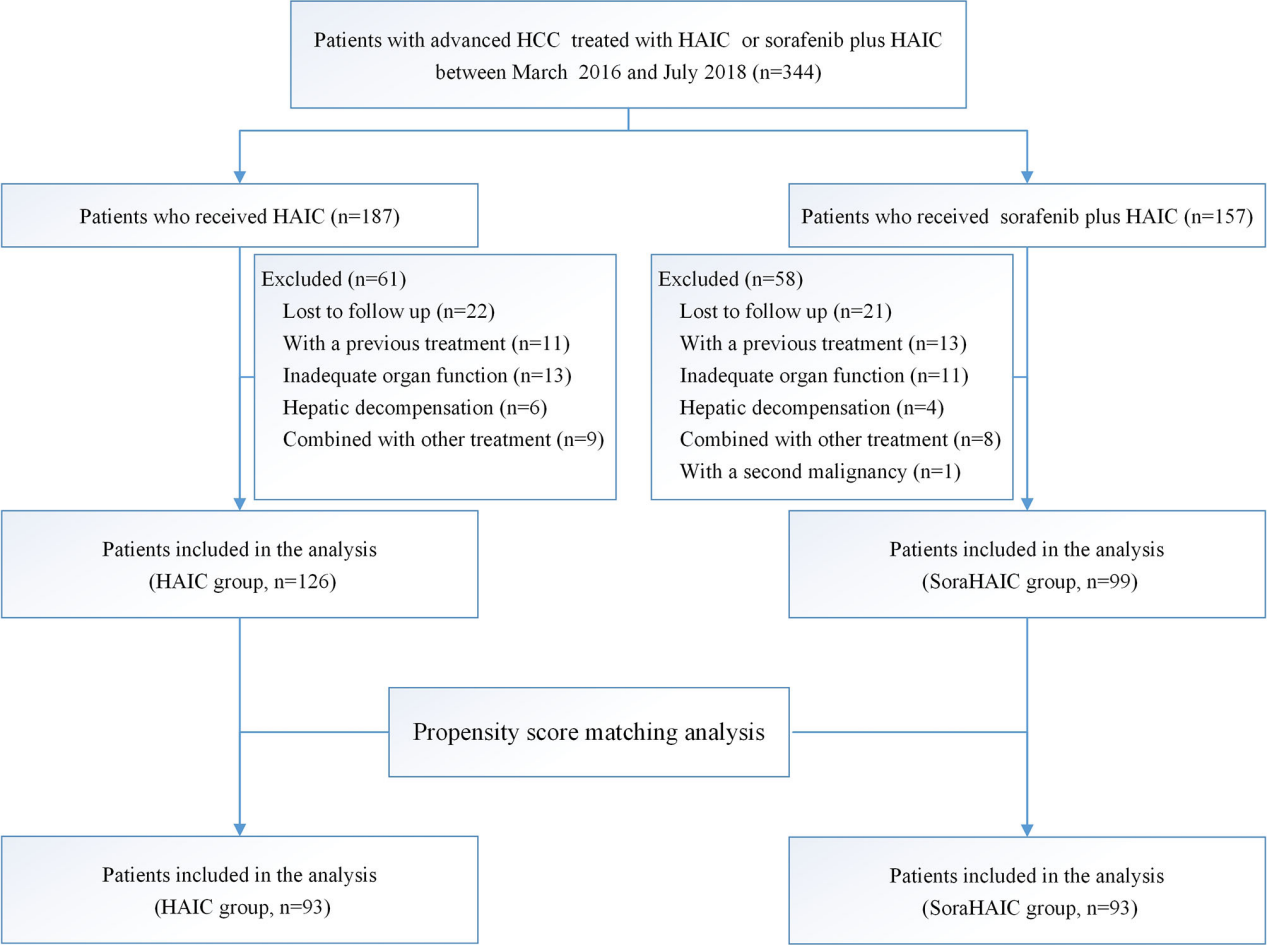
Flowchart showing the patient selection process. HAIC, hepatic arterial infusion chemotherapy of oxaliplatin, 5-fluorouracil and leucovorin; soraHAIC, hepatic arterial infusion chemotherapy of oxaliplatin, 5-fluorouracil and leucovorin plus sorafenib.

The inclusion and exclusion process used in this study is shown in **Figure 1**. Ultimately, 225 patients were included in this study.

### Treatment Option

When advanced HCC was confirmed, the treatment of HAIC using FOLFOX alone and HAIC plus sorafenib as initial treatments were both recommended to the patients. Each patient was informed of the efficacy and safety of the treatment of HAIC using FOLFOX alone or HAIC plus sorafenib before they made their choices according to previous studies (22–26).

### HAIC Alone Cohort

In the HAIC alone group, HAIC was repeated every 3 weeks and the interruptions and dose reductions were the same as reported in our previous studies (11, 27). One of 5 doctors (M.K.H, Q.J.L, W.W, Y.J.Z, M.S, with 5, 9, 15, 12, and 21 years of experience performing transarterial chemoembolization, respectively) performed HAIC according to the following protocol: a catheter was inserted into the truncus celiacus or superior mesenteric artery for arteriography. Then, the tip of a microcatheter was superselectively inserted and located in the main feeding hepatic artery depending on the arterial supply of the tumor. The other end of microcatheter was marked and fixed *in vitro* and connected to the artery infusion pump to administer the chemotherapy agent: oxaliplatin 85 mg/m^2^ from hour 0 to 2 on day 1; leucovorin 400 mg/m^2^ from hour 2 to 3 on day 1; 5-fluorouracil 400 mg/m^2^ bolus at hour 3 and 2400 mg/m^2^ over 46 hours on days 1 and 2. When the mark moved, bedside X-ray radiography was also conducted to confirm the location of the catheter tip. If dislocation of the catheter tip was confirmed, the patient was transferred to the digital subtraction angiography room to correct the location of the catheter tip. After the regimen was completed, the catheter was removed.

### HAIC Plus Sorafenib (soraHAIC) Cohort

Patients were treated by HAIC as described above. In addition, these patients were also treated with 400 mg sorafenib twice daily. Sorafenib interruptions and dose reductions were based on a previous study (5). If a patient could not tolerate the lowest dose, sorafenib would be discontinued. Sorafenib was allowed to begin before or after HAIC, but the start time of these two treatments was within a week.

### Follow-Up and Assessments

Treatments were maintained until one of the following situations occurred: tumor progression; intolerable toxicity; the need for surgery, ablation, or transarterial chemoembolization owing to downstaging; or at the patient’s request. After tumor progression, subsequent treatments would be recommended by the doctors and finally decided by the patients. The follow up and assessments were carried out in the same manner as that in prior trial (11). The follow-up ended on September 3, 2019. Before HAIC treatment was discontinued, a blood examination and safety assessment were conducted every HAIC cycle. Additionally, upper abdomen-enhanced CT/MRI and chest-enhanced CT were performed every 6 (± 1) weeks. Tumor assessments were retrospectively evaluated by 2 independent doctors who were blinded to the treatment groups according to RECIST 1.1 with 4 levels: complete response (CR), partial response (PR), stable disease (SD) and progressive disease (PD). If there was a controversy in the tumor assessments, the final judgment was made by another more experienced radiologist.

Overall survival (OS) was calculated from the date of the start of HAIC to death from any cause or the date of the last follow-up. Progression-free survival (PFS) was calculated from the date of the start of HAIC to progression according to RECIST 1.1 criteria or death from any cause, whichever occurred first. The disease control rate (DCR) was the percentage of patients who achieved complete response, partial response or stable disease, and the objective response rate (ORR) was the percentage of patients who achieved complete response or partial response based on RECIST 1.1 (20). Adverse events were assessed by the National Cancer Institute Common Terminology Criteria for Adverse Events version 4.03.

### Statistical Analysis

Student’s t tests or chi-square tests were used to compare the results. The Kaplan-Meier method and log-rank test were used to compare survival outcomes. A multivariable Cox proportional hazards model was used to analyze factors with *P* < 0.10 using a univariate analysis. *P* < 0.05 was considered significant, and Statistical Package for Social Sciences (SPSS, version 24; IBM, Armonk, NY) was used to perform analyses.

Propensity score matching analysis was used to reduce the impact of selection bias and potential confounding factors between the groups. The data after propensity score matching analysis formed the propensity-score-matched cohort. To reduce the impact of selection bias as much as possible, 10 clinical parameters were included in the propensity score matching analysis, including age, gender, ECOG-PS score, tumor size, tumor number, portal vein tumor thrombus, hepatic vein tumor thrombus, AFP, albumin, and extrahepatic metastasis. Matched pairs were then formed using a one‐to‐one nearest‐neighbor caliper of width 0.2.

## Results

From March 1, 2016 to July 22, 2018, 344 consecutive patients with advanced HCC were treated with either HAIC alone or HAIC plus sorafenib. Ultimately, 225 patients were included in this study: 126 received the treatment of HAIC alone and 99 received HAIC plus sorafenib (**Figure 1**). Patient demographics was shown in **Table 1**. No difference was observed in the baseline characteristics of the original cohort. Using the propensity-score matching method, we obtained the one-to-one paired cohort (93 patients in each group). The baseline characteristics were well balanced in the PSM cohort too (**Table 1**).

**Table 1:** Patient baseline demographic and clinical characteristics.

	Initial cohort			Propensity-score-matched cohort		
	HAIC (n=126)	HAIC + sorafenib (n = 99)	*P*	HAIC (n = 93)	HAIC + sorafenib (n = 93)	*P*
Age			0.25			0.46
≤50	68 (54.0%)	61 (61.6%)		53 (57.0%)	58 (62.4%)	
>50	58 (46.0%)	38 (38.4%)		40 (43.0%)	35 (37.6%)	
Gender			0.23			0.60
male	111 (88.1%)	92 (92.9%)		84 (90.3%)	86 (92.5%)	
female	15 (11.9%)	7 (7.1%)		9 (9.7%)	7 (7.5%)	
ECOG			0.43			0.43
0-1	89 (70.6%)	65 (65.7%)		66 (71.0%)	61 (65.6%)	
2	37 (29.4%)	34 (34.3%)		27 (29.0%)	32 (34.4%)	
AFP			0.35			0.87
≤400	39 (31.0%)	25 (25.3%)		24 (25.8%)	23 (24.7%)	
>400	87 (69.0%)	74 (74.7%)		69 (74.2%)	70 (75.3%)	
Tumor Size			0.67			0.77
≤10	60 (47.6%)	50 (50.5%)		46 (49.5%)	48 (51.6%)	
>10	66 (52.4%)	49 (49.5%)		47 (50.5%)	45 (48.4%)	
Tumor Number			0.07			0.43
≤3	36 (28.6%)	18 (18.2%)		13 (14.0%)	17 (18.3%)	
>3	90 (71.4%)	81 (81.8%)		80 (86.0%)	76 (81.7%)	
PVTT^†^			0.37			0.76
Vp 0-2	52 (41.3%)	35 (35.4%)		33 (35.5%)	35 (37.6%)	
Vp 3-4	74 (58.7%)	64 (64.6%)		60 (64.5%)	58 (62.4%)	
HVTT			0.58			0.74
No	92 (73.0%)	69 (69.7%)		68 (73.1%)	66 (71.0%)	
Yes	34 (27.0%)	30 (30.3%)		25 (26.9%)	27 (29.0%)	
Metastasis			0.87			0.55
No	75 (59.5%)	60 (60.6%)		58 (62.4%)	54 (58.1%)	
Yes	51 (40.5%)	39 (39.4%)		35 (37.6%)	39 (41.9%)	
Lung only	8 (6.3%)	10 (10.1%)		5 (5.4%)	10 (10.1%)	
Lymph node only	23 (18.3%)	11 (11.1%)		15 (16.1%)	11 (11.1%)	
Bone only	2 (1.6%)	2 (2.0%)		1 (1.1%)	2 (2.0%)	
Adrenal gland only	2 (1.6%)	2 (2.0%)		2 (2.1%)	2 (2.0%)	
Peritoneal implantation only	1 (0.8%)	0 (0.0%)		1 (1.1%)	0 (0.0%)	
Multiple organs	15 (11.9%)	14 (14.2%)		11 (11.8%)	14 (14.2%)	
ALB			0.50			0.77
<40	63 (50.0%)	54 (54.5%)		49 (52.7%)	51 (54.8%)	
≥40	63 (50.0%)	45 (45.5%)		44 (47.3%)	42 (45.2%)	
HBV infection			0.52			0.23
Yes	117 (92.9%)	94 (94.9%)		85 (91.4%)	89 (95.7%)	
No	9 (7.1%)	5 (5.1%)		8 (8.6%)	4 (4.3%)	
ALT			0.30			0.38
≤45	66 (52.4%)	45 (45.5%)		47 (50.5%)	41 (44.1%)	
> 45	60 (47.6%)	54 (54.5%)		46 (49.5%)	52 (55.9%)	
AST			0.48			0.55
≤60	53 (42.1%)	37 (37.4%)		37 (39.8%)	33 (35.5%)	
>60	73 (57.9%)	62 (62.6%)		56 (60.2%)	60 (64.5%)	
TBil			0.14			0.21
≤20	93 (73.8%)	64 (64.6%)		67 (72.0%)	59 (63.4%)	
>20	33 (26.2%)	35 (35.4%)		26 (28.0%)	34 (36.6%)	

Data are n (%). soraHAIC group, sorafenib plus hepatic arterial infusion chemotherapy of oxaliplatin, 5-fluorouracil and leucovorin group.

HAIC group, hepatic arterial infusion chemotherapy of oxaliplatin, 5-fluorouracil and leucovorin group.

ECOG, Eastern Cooperative Oncology Group. HBV, hepatitis B virus. AFP, alpha fetoprotein. ALT, Alanine aminotransferase.

AST, Aspartate aminotransferase; TBil, total bilirubin; Alb, albumin; PVTT, portal vein tumor thrombus. HVTT, hepatic vein tumor thrombus.

^†^PVTT was according to Liver Cancer Study Group of Japan criteria. Vp0 indicates no portal vein invasion, Vp1 third branch portal vein invasion, Vp2 second branch portal vein invasion (segmental invasion), Vp3 first branch portal vein invasion (branch invasion), and Vp4 main portal vein invasion. P value was calculated by chi-square tests.

The patients received a total of 744 cycles of HAIC therapy. The mean and median number of HAIC administrations in the soraHAIC group were 3.3 [SD=1.68] and 3 [IQR 2-4], and those in the HAIC group were 3.31 [SD=1.86] and 3 [IQR 2-4]. The median dose intensity of sorafenib (range) was 612 mg/day (200–800 mg) in the soraHAIC group. After tumor progression, 52 patients in the soraHAIC group and 70 patients in the HAIC group underwent subsequent treatment, including transarterial chemoembolization (soraHAIC: n=14; HAIC: n=12), resection (soraHAIC: n=7; HAIC: n=8), lenvatinib (soraHAIC: n=14; HAIC: 11), sorafenib (soraHAIC: n=0; HAIC: n=37), immune checkpoint inhibitors (soraHAIC: n=8; HAIC: n=9), radiotherapy (soraHAIC: n=6; HAIC: n=8), systemic chemotherapy (soraHAIC: n=5; HAIC: n=7), and ablation (soraHAIC: n=6; HAIC: n=5). There was also no difference in the subsequent treatments between the two groups except that more patients (n=37) in the HAIC group received sorafenib. The median OS and PFS of patients who received additional sorafenib treatment after tumor progression in the HAIC group was 11.87 months (95% CI, 10.24-13.5) and 6.77 months (95% CI, 5.58-7.96).

**Figure 2:**
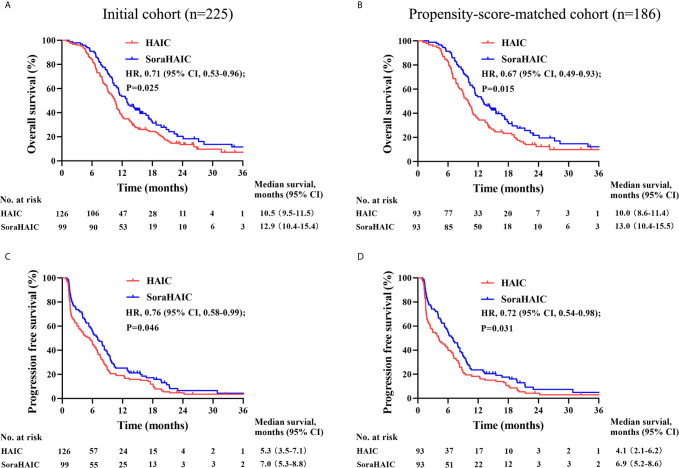
Kaplan-Meier curves for overall survival **(A)** and progression-free survival **(C)** in the initial cohort and overall survival **(B)** and progression-free survival **(D)** in the propensity-score-matched cohort.

For the original cohort, 185 patients had died at the time of analysis (110 patients in the HAIC group and 75 patients in the soraHAIC group). The median OS in the soraHAIC group was 12.9 months (95% CI, 10.4-15.4) compared with 10.5 months (95% CI, 9.5-11.5) in the HAIC alone group (HR=0.71; 95% CI, 0.53-0.96; *P*=0.025; **Figure 2A**). The median PFS in the soraHAIC group was 7.0 months (95% CI, 5.3-8.8) compared with 5.3 months (95% CI, 3.5-7.1) in the HAIC alone group (HR=0.76; 95% CI, 0.58-0.99; *P*=0.046; **Figure 2C**). The results of univariable and multivariable analyses of overall survival were listed in **Table 2**. Multivariable analysis showed that independent risk factors for survival were type of treatment (soraHAIC vs. HAIC alone, HR=0.69; 95% CI, 0.51-0.93; *P*=0.013), portal vein tumor thrombus (PVTT grade Vp3-4 vs. Vp0-2, HR=1.54; 95% CI, 1.12-2.11; *P*=0.007), and extrahepatic metastasis (presence vs. absence, HR=1.71; 95% CI, 1.25-2.34; *P*=0.001, **Table 2**). In addition, the DCR was significantly higher in the soraHAIC group than in the HAIC alone group (74.8% vs. 61.1%, *P*=0.03), while the ORR was similar (37.4% vs. 36.5%, *P*=0.89) in the two groups based on the RECIST 1.1 criteria (**Table 3**).

**Table 2:** Univariate and multivariate analysis of overall survival in initial and propensity-score-matched cohorts.

	Initial cohort			Propensity-score-matched cohort (1:1)		
	Univariate analysis	Multivariate analysis		Univariate analysis	Multivariate analysis	
	*P1*	HR (95% CI)	*P2*	*P1*	HR	*P2*
Group (HAIC/soraHAIC)	0.026	0.69 (0.51-0.93)	0.013	0.016	0.65 (0.47-0.90)	0.009
Age, year (≤50/>50)	0.99			0.86		
Gender (male/female)	0.84			0.60		
ECOG (0-1/2)	0.034	1.18 (0.86-1.62)	0.30	0.041	1.25 (0.88-1.76)	0.21
Tumor size, cm (≤10/>10)	0.16			0.083	1.22 (0.88-1.70)	0.24
Tumor number (≤3/>3)	0.30			0.17		
PVTT (Vp0-2/Vp3-4)	0.055	1.54 (1.12-2.11)	0.007	0.060	1.45 (1.02-2.07)	0.041
HVTT (no/yes)	0.64			0.49		
Metastasis (no/yes)	0.002	1.71 (1.25-2.34)	0.001	0.009	1.66 (1.18-2.32)	0.004
AFP, ng/ml (≤400/>400)	0.11			0.29		
ALB, g/L (≤40/>40)	0.035	0.77 (0.58-1.04)	0.08	0.038	0.74 (0.53-1.02)	0.07

soraHAIC, sorafenib plus hepatic arterial infusion chemotherapy of oxaliplatin, 5-fluorouracil and leucovorin. HAIC= hepatic arterial infusion chemotherapy of oxaliplatin, 5-fluorouracil and leucovorin; HR, hazard ratio; CI, confidence interval; ECOG, Eastern Cooperative Oncology Group; PVTT, portal vein tumor thrombus; HVTT, hepatic vein tumor thrombus. HBsAg, hepatitis B surface antigen; PVTT, portal vein tumor thrombus; ALT, Alanine aminotransferase; AST, Aspartate aminotransferase; Alb, albumin; TBIL, total bilirubin; AFP. alpha fetoprotein.

P1 value was calculated with two-sided log-rank test. Any factors that were statistically significant at P less than 10% in the univariate analysis were candidates for entry into a multivariable Cox analysis.

P2 value was calculated by multivariable Cox proportional-hazards analysis.

**Table 3:** Summary of best response based on the RECIST criteria.

	Overall response (before PSM)			Overall response (after PSM)		
	HAIC group (%)	SoraHAIC group (%)	*P*	HAIC group (%)	SoraHAIC group (%)	*P*
CR	0 (0)	0 (0)		0 (0)	0 (0)	
PR	46 (36.5)	37 (37.4)	0.89	31 (33.3)	34 (36.6)	0.64
SD	31 (24.6)	37 (37.4)	0.04	21 (22.6)	35 (37.6)	0.03
PD	49 (38.9)	25 (25.2)	0.03	41 (44.1)	24 (25.8)	0.01
DCR	77 (61.1)	74 (74.8)	0.03	52 (55.9)	69 (74.2)	0.01
ORR	46 (36.5)	37 (37.4)	0.89	31 (33.3)	34 (36.6)	0.88

RRECIST, Response Evaluation Criteria in Solid Tumors; CR, complete response; PR, partial response; SD, stable disease; PD, progressive disease; DCR, disease control rate; ORR, objective response rate.

SoraHAIC group, sorafenib plus hepatic arterial infusion chemotherapy group, Sorafenib group, sorafenib monotherapy group.

For the propensity-score-matched cohort, the median overall survival in the soraHAIC group was 13.0 months (95% CI, 10.4-15.5) compared with 10.0 months (95% CI, 8.6-11.4) in the HAIC group (HR=0.67; 95% CI, 0.49-0.93; *P*=0.015; **Figure 2B**). The median progression-free survival in the soraHAIC group was 6.9 months (95% CI, 5.2-8.6) compared with 4.1 months (95% CI, 2.1-6.2) in the HAIC alone group (HR 0.72 [95% CI, 0.54-0.98]; *P*=0.031; **Figure 2D**). Multivariable analysis showed that independent risk factors for survival were type of treatment (soraHAIC vs. HAIC, HR=0.65; 95% CI, 0.47-0.90; *P*=0.009), portal vein tumor thrombus (PVTT grade Vp3-4 vs. Vp0-2, HR=1.45; 95% CI, 1.02-2.07; *P*=0.041), and extrahepatic metastasis (presence or absence, HR=1.66; 95% CI, 1.18-2.32; *P*=0.004) (**Table 2**). As predicted, the DCR was significantly higher in the soraHAIC group than in the HAIC group (74.2% vs. 55.9%, *P*=0.01), while the ORR was similar (36.6% vs. 33.3%, *P*=0.88) in the two groups based on the RECIST 1.1 criteria in the propensity-score-matched cohort (**Table 3**).

The treatment-related adverse events with high incidence rates (≥10%) are shown in **Table 4**. The frequencies of all-grade hand–foot skin reaction, rash, vomiting, diarrhea and nausea were significantly higher in the soraHAIC group than in the HAIC group (*P*<0.05). Grade 3–4 hand–foot skin reaction was more frequent in the soraHAIC group (*P*<0.001). Serious adverse events occurred in 10 (10.1%) of 99 patients (1 hepatic encephalopathy, 3 gastrointestinal bleeding, 2 renal failure, and 4 ascites) who received HAIC plus sorafenib, and 11 (8.7%) of 126 patients who received HAIC (5 gastrointestinal bleeding, 3 renal failure, and 3 ascites) (*P*=0.73). No treatment-related deaths were observed within one month of the initial treatment in each group. There was no difference in the reduction (36 of 99 patients vs. 42 of 126 patients, *P*=0.64), delay (25 of 99 patients vs. 26 of 126 patients, *P*=0.41), or discontinuation (30 of 99 patients vs. 33 of 126 patients, *P*=0.50) of HAIC treatment because of adverse events between the two groups.

**Table 4:** Treatment Related Adverse Events^†^.

Adverse event	HAIC group (n=126)		SoraHAIC group (n=99)		*P* value	
	Any grade (%)	Grade 3-4 (%)	Any grade (%)	Grade 3-4 (%)	Any grade	Grade 3-4
Overall incidence	112 (88.9)	50 (39.7)	93 (93.9)	51 (51.5)	0.19	0.08
Blood/bone marrow suppression						
Neutropenia	38 (30.2)	5 (4)	40 (40.4)	8 (8.1)	0.11	0.19
Thrombocytopenia	55 (43.7)	7 (5.6)	52 (52.5)	10 (10.1)	0.19	0.2
Anemia	68 (54)	5 (4)	62 (62.6)	6 (6.1)	0.19	0.47
Constitutional symptoms						
Fatigue	82 (65.1)	5 (4)	75 (75.8)	6 (6.1)	0.08	0.47
Fever	11 (8.7)	0	13 (13.1)	0	0.29	–
Weight loss	42 (33.3)	2 (1.6)	41 (41.4)	2 (2)	0.21	0.81
Dermatologic events						
Hand–foot skin reaction	0	0	46 (46.5)	12 (12.1)	<0.001	<0.001
Alopecia	10 (7.9)	0	12 (12.1)	0	0.29	–
Rash	7 (5.6)	0	16 (16.2)	0	0.01	–
Gastrointestinal events						
Nausea	60 (47.6)	6 (4.8)	74 (74.7)	8 (8.1)	<0.001	0.31
Vomiting	58 (46)	8 (6.3)	63 (63.6)	8 (8.1)	0.01	0.55
Diarrhea	20 (15.9)	5 (4)	30 (30.3)	7 (7.1)	0.01	0.3
Abdominal pain	45 (35.7)	6 (4.8)	38 (38.4)	5 (5.1)	0.68	0.92
Neurotoxicity						
Sensory neuropathy	47 (37.3)	0	39 (39.4)	0	0.75	–
Hepatic function						
Elevated ALT	90 (71.4)	20 (15.9)	79 (79.8)	14 (14.1)	0.15	0.72
Elevated AST	96 (76.2)	28 (22.2)	85 (85.9)	18 (18.2)	0.07	0.46
Hyperbilirubinemia	74 (58.7)	8 (6.3)	64 (64.6)	7 (7.1)	0.37	0.83
Hypoalbuminemia	88 (69.8)	5 (4)	75 (75.8)	3 (3)	0.32	1

ALT, alanine aminotransferase; AST, aspartate aminotransferase.

soraHAIC group, sorafenib plus hepatic arterial infusion chemotherapy group; HAIC group, hepatic arterial infusion chemotherapy group.

P value was calculated by a two-sided chi-square test.

^†^Listed are adverse events, as defined by the National Cancer Institute Common Terminology Criteria (version 4.03), that occurred in at least 10% of patients in either study group.

## Discussion

In the present study, we compared the efficacy and safety of HAIC using FOLFOX plus sorafenib to HAIC alone as initial treatment for patients with advanced HCC. We found that the HAIC using FOLFOX plus sorafenib group presented a longer OS (12.9 vs. 10.5 months, *P*=0.025), a better PFS (7.0 vs. 5.3 months, *P*=0.046), and a higher disease control rate (RECIST 1.1: 74.8% vs. 61.1%, *P*=0.030) than the HAIC alone group. Multivariate analysis suggested that the treatment of HAIC using FOLFOX plus sorafenib was an independent favor factors for OS compared with HAIC alone in advanced HCC. In terms of safety, both HAIC using FOLFOX plus sorafenib and HAIC alone had acceptable safety profiles. Similar results were found in the propensity score matching cohort. Based on these findings, it is suggested that HAIC using FOLFOX plus sorafenib may be superior to HAIC alone as initial treatment for advanced HCC.

Previous studies have shown that both HAIC plus sorafenib and HAIC monotherapy can significantly improve survival compared with sorafenib (10, 11). However, it is still unclear whether the prolonged survival benefit should be attributed to the synergism between HAIC with FOLFOX and sorafenib or just HAIC alone. In this study, the OS, PFS and tumor response in the soraHAIC group were consistent with those in prior studies (11, 26). The OS in the HAIC alone group was lower than that in the previous study (10). This finding might be explained by the fact that more patients in this study had PVTT and increased concentrations of AFP compared with the patients in the previous study (10). And these characteristics (PVTT and increased concentrations of AFP) have been shown to be adverse prognostic factors for mortality in patients with advanced HCC (26, 28). The present study indicated that HAIC using FOLFOX plus sorafenib may improve the OS and PFS compared with HAIC alone. The survival difference between HAIC plus sorafenib and HAIC alone suggested that the extra survival benefit may be partly due to the synergistic antitumor effect of sorafenib and HAIC with sorafenib extending survival through disease stabilization and HAIC shrinking tumors (29).

In addition, a higher disease control rate was observed in the soraHAIC group (74.8% vs. 61.1%, *P*=0.030). It seems that the addition of sorafenib to HAIC using FOLFOX could delay disease progression but does not improve the rates of partial response compared with HAIC alone. This finding might be related to the disease stabilization of sorafenib (5). Previous studies also showed that patients who were treated with sorafenib had a low partial response rate (2%-3.3%) and high stable disease rate (54%-71%) (5, 6). Moreover, the soraHAIC group had significantly elevated frequencies of all-grade hand–foot skin reaction, rash, vomiting, diarrhea, nausea and grade 3-4 hand–foot skin reaction, these complications were also common in sorafenib monotherapy in the previous studies (5, 6). Therefore, the adverse events were not unexpected and were manageable by expectant treatment, treatment interruption or dose modification.

There were some limitations in this study. First, this was a retrospective study, which was affected by baseline confounding factors. To improve the intergroup comparability, propensity score matching analysis was used, and the baseline characteristics were well balanced. Second, more than 90% of the patients had hepatitis B virus infection. As such, whether these findings may be applicable to Western countries, where HCC is more commonly caused by hepatitis C virus and alcohol use (30), needs further study. Finally, subsequent treatments might have an impact on the OS of patients. However, there was no difference in the subsequent treatments between the two cohorts except that some patients in the HAIC alone group received subsequent sorafenib which might improve the OS and PFS of patients in the HAIC alone group.

In summary, HAIC using FOLFOX plus sorafenib may improve OS, PFS and the disease control rate compared with HAIC alone in patients with advanced HCC. HAIC using FOLFOX plus sorafenib may be superior to HAIC alone as initial treatment for advanced HCC. A large-sample, prospective, randomized controlled trial is needed to compare HAIC using FOLFOX plus sorafenib with HAIC alone for advanced HCC.

## Data Availability Statement

The raw data supporting the conclusions of this article will be made available by the authors, without undue reservation.

## Ethics Statement

The studies involving human participants were reviewed and approved by the Institutional Review Board of Sun Yat-sen University Cancer Center. The patients/participants provided their written informed consent to participate in this study.

## Author Contributions

MS: Study conception. QJL and MS: Study design. R-BL, YZ, M-KH, D-SW, X-YB, Y-XH, Z-CL, Y-JX, AK, WW, Y-JZ, M-SC, R-PG, Q-JL, and MS: Acquisition of data. R-BL, M-KH, D-SW, X-YB, Y-XH, and AK: Analysis and interpretation of data. All authors contributed to the article and approved the submitted version.

## Conflict of Interest

The authors declare that the research was conducted in the absence of any commercial or financial relationships that could be construed as a potential conflict of interest.

## Abbreviations

AEs, adverse events; AFP, alpha-fetoprotein; CT, computed tomography; CI, confidence interval; ECOG PS, Eastern Cooperative Oncology Group performance status; FOLFOX, oxaliplatin, fluorouracil, and leucovorin; HCC, hepatocellular carcinoma; HAIC, hepatic arterial infusion chemotherapy; HR, hazard ratio; PVTT, portal vein tumor thrombus; RECIST, Response Evaluation Criteria in Solid Tumors.
